# General surface circulation controls the interannual fluctuations of anchovy stock biomass in the Central Mediterranean Sea

**DOI:** 10.1038/s41598-020-58028-0

**Published:** 2020-01-31

**Authors:** Bernardo Patti, Marco Torri, Angela Cuttitta

**Affiliations:** 1 National Research Council of Italy (CNR), Institute of Anthropic Impacts and Sustainability in marine environment (IAS), unit of Palermo, Palermo (PA), Italy; 2 National Research Council of Italy (CNR), Institute of Anthropic Impacts and Sustainability in marine environment (IAS), unit of Capo Granitola, Campobello di Mazara (TP), Italy; 3 National Research Council (CNR), Institute for studies on the Mediterranean (ISMed), unit of Palermo, Palermo (PA), Italy

**Keywords:** Marine biology, Physical oceanography

## Abstract

The sustainable exploitation of small pelagic fish populations, characterized by short life span and early age at first reproduction, is typically more influenced by the success of annual recruitment rather than by fishing mortality. Recruitment strength, in turn, is related to the high environmental variability characterizing the pelagic fish habitats, able to strongly affect the survival of early stages, from hatching to recruitment. Here, we consider the case study of anchovy (*Engraulis encrasicolus*) stock in the Strait of Sicily (Central Mediterranean). The interannual fluctuations exhibited over an 18-year long period by this fish population was found to be mainly linked to surface circulation patterns, as far as they are able to control retention/dispersal processes of larval stages. We firstly used Lagrangian simulations to reproduce the fate of anchovy early stages during their planktonic phase. Larval retention indices constructed from the output of the simulations were able alone to explain a large proportion of variance (up to 70%) in yearly biomass of the anchovy population, outclassing the other environmental factors considered in this study. Such results are relevant for fisheries management, for all fish stocks characterized by potentially high vulnerability of early life stages.

## Introduction

The importance of environmental forcings as drivers for the interannual variability in the standing stock biomass of small pelagic fish species has been well documented in several papers^1–3^. In general, this applies to r-strategist fish species, characterized by short life span, early age at first reproduction, high number of offspring and low offspring survival rates, which in turn can heavily affect recruitment success and stock biomass levels. Conversely, effects of variations in fishing pressure may have a limited impact on the recruitment and on the standing stock biomass^4–6^. This pattern is enhanced in highly variable marine environments such as the Northern side of the Strait of Sicily (SoS).

Here, surface circulation is dominated by the flow of the Modified Atlantic Waters, locally named Atlantic Ionian Stream (AIS)^7^. The role of AIS in mimicking the “fundamental triad” of factors underlying favorable fish reproductive habitats^8^, i.e. (i) nutrient enrichment, (ii) concentration of larval food distributions, and (iii) local retention of eggs and larvae, has been reported for the SoS in many papers so far^9–11^.

Actually, the study area is characterized by strong coastal upwelling events, which are able to enrich the typically oligotrophic Mediterranean waters^12–14^. However, the advection of surface waters from coastal areas may be detrimental for the survival of larval stages due to food availability constraints characterizing the offshore environment^15–17^.

The hydrographic circulation in the Northern side of the SoS affects the distribution of eggs and larval stages of the European anchovy (*Engraulis encrasicolus*, Linnaeus, 1758), and it was also found to be potentially relevant for the success of the yearly recruitment of this fish resource^17^. Actually, the fate of early larval stages under the effect of local surface circulation during the summer (spawning season) is able to impact on the larval survival rates, depending on food availability differences between inshore/offshore areas.

The role of hydrodynamic processes on anchovy eggs and larvae distribution in Northern SoS was firstly investigated by Lagrangian numerical simulations using as Eulerian input the velocity fields provided by the Mediterranean Sea Forecasting System (MFS), taking into account the small-scale 2D and 3D dynamics^18–20^. This approach confirmed previous empirical observations about the impact of the wind-induced coastal current in transporting anchovy larvae from spawning areas to the recruiting area off the Sicilian south-eastern tip^12,16,21^. In addition, it also evidenced that the significant cross-shore transport, induced by the combination of strong northwesterly mistral winds and topographic effects, is able to deliver larvae away from the coastal “conveyor belt”.

Previous studies about the potential spawning habitat of anchovy stock in the study area also showed the high interannual variability characterizing the distribution patterns of the main anchovy spawning grounds over the continental shelf of the study area^22,23^.

Anchovy spawning activity in the study area spans from April to October but is more intense during the summer, with a peak in June-July^24^. On the other hand, surface circulation is quite variable during the anchovy spawning period in the study area^25^. An obvious consequence is that the fate of anchovy offspring is expected to be highly variable due to the very different environmental conditions characterizing the areas where larvae are advected from hatching up to the end of their planktonic life.

The importance of environmental forcings in controlling the dynamics of anchovy population is also indirectly confirmed by the very high level of biomass fluctuation, which locally appears to be largely unrelated to the levels of fishing pressure^26–28^.

The basic assumption of this paper is that the higher the proportion of post-larval specimens retained in a certain year in areas favorable for their survival (i.e. shelf areas, characterized by higher chlorophyll-a concentrations compared to offshore areas), the greater the probability of these retention processes in contributing to a strong yearly recruitment the following year. Considering that the bulk of anchovy stock biomass off the southern coast of Sicily (FAO Geographical Sub-Area 16, GSA16) is represented by age class 1^29^, information about larval retention is expected to be relevant for inferring predictions on the anchovy standing stock biomass.

An ancillary hypothesis tested in this paper is whether information about the variable locations across years of the main anchovy spawning grounds, as obtained by the ichthyoplankton surveys carried out from 1997 to 2013, may be relevant for obtaining more accurate biomass predictions of the anchovy population than considering a uniform distribution for its spawning activity. This hypothesis was tested using two different approaches for the releasing sites of particles representing larval specimens in the Lagrangian simulation runs, corresponding to the two assumed spawning patterns. To this aim, information on the anchovy potential spawning habitat as determined by a previous study was also used^22^.

Finally, other environmental data (namely, the average sea surface temperature and chlorophyll-a concentration over the shelf areas of GSA 16 during the anchovy spawning period and during the pre-juvenile development period) were also incorporated in a multifactorial modelling approach, in order to test their possible role in improving the ability of model predictions.

## Materials and Methods

### Anchovy standing stock biomass estimates

Hydro-acoustic biomass estimates of the anchovy population over the period 1998–2014 are available for the study area^28^. These biomass estimates were used as response variable in different linear and non-linear statistical modelling approaches. The aim was to investigate the relationship between a proxy for larval and post-larval retention (and enhanced survival) at time (t) and the strength of the anchovy stock recruitment to local fisheries the following year, at time (t + 1). The choice of using total anchovy biomass as a proxy of the yearly recruitment is supported by the available information on the age structure of anchovy population during the summer. In general, during the summer age classes 1–2 were shown to dominate the yearly anchovy stock biomass^29,30^. Over the period 2009–2014, ages 1–2 account for 81.2% in numbers and 91.0% in weight of the whole population, with age class 1 alone representing more than 2/3 of total biomass^28,31^. So, each one of the yearly estimates of anchovy biomass is mostly the result of the success of the recruitment processes characterizing the stock from the spawning period of the previous year onwards.

### Anchovy catch data

Over the period 1998–2014, average anchovy landings in Sciacca port were about 2,000 metric tons, with large interannual fluctuations unrelated to changes in the anchovy standing stock biomass, whereas fishing effort (fishing days) remained quite stable^26^. For each year over period 1998–2014, total landings (in tons) comprised between July at year (t) and June at year (t + 1), labelled as “mid-year landings”, were used to build the time series “biomass + catch”. This series represents the acoustic biomass estimates at year (t) integrated by the “mid-year landings”, which approximately correspond to the fish production between two consecutive summer acoustic surveys. This new variable along with the standard yearly acoustic stock biomass estimate were used as dependent variables in the statistical modelling approaches, in order to explore their relationships with the larval retention indices as defined below. The amount of these mid-year landings in the considered period was on average about 40% of the stock biomass estimated from the summer scientific surveys.

### Plankton sampling

Plankton samples were collected using a bongo net (40-cm opening) towed obliquely from the surface to a 100-m depth, equipped with a 200-µm mesh size net.

Samples were immediately fixed after collection and preserved in a 10% buffered-formaldehyde (and/or 70% alcohol) and sea-water solution for further sorting in laboratory by stereomicroscopy. For each sampling station, the resulting counts of anchovy eggs were standardized to numbers per cubic meter using the volume measurements of filtered sea-water, obtained by mechanical flowmeters (General Oceanics Inc., FL, USA).

**Figure 1:**
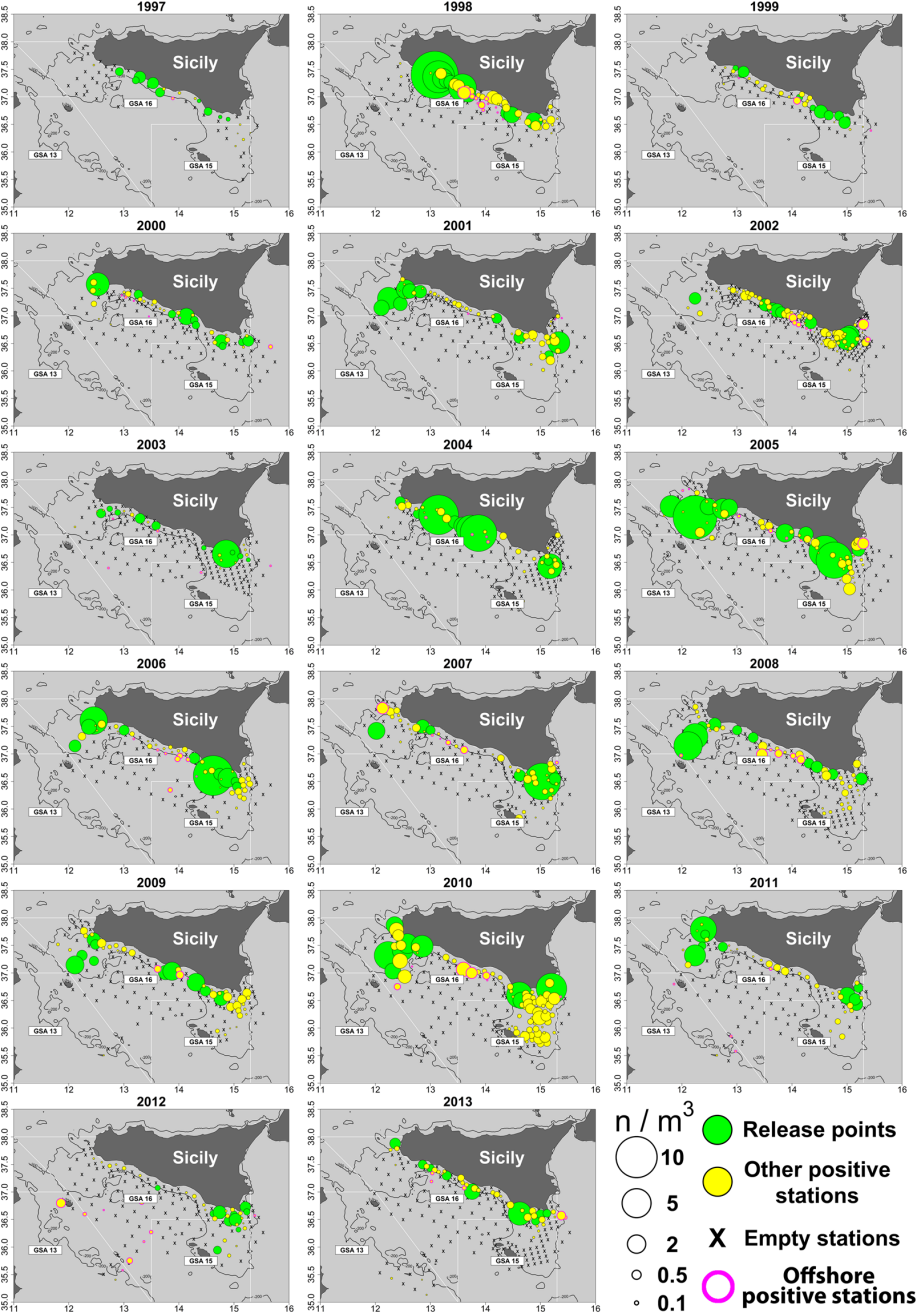
Anchovy egg distributions in the summer surveys carried out in the study area from 1997 to 2013. Circle sizes are proportional to egg concentration. In green the locations of the top 10 stations over the continental shelf areas in terms of anchovy eggs densities, by year, used as releasing points in the Lagrangian simulation runs; in yellow the other positive stations for the presence of anchovy eggs in the samples. Sampling stations are indicated using “x” symbol. Continental shelf bathymetry (200 m depth) is also depicted with a continuous black line.

For each one of the yearly summer ichthyoplankton surveys considered in this study (1997–2013), having a typical duration of about 2–3 weeks, the geographical coordinates of the stations over the continental shelf (bottom depth < 200 m) scoring the top ten highest anchovy egg density were selected as starting points for the Lagrangian simulation runs (see Fig. 1).

### Transport model

The advection of Lagrangian elements (particles) released at different sites and dates in the study area, representing anchovy eggs and developing larvae^32–34^, were simulated using the GNOME software package^35–37^. In this study, the main Eulerian input for Lagrangian simulation runs were from daily satellite-based estimates of the surface current velocity fields. In addition, the effect of wind on the sea surface was also considered as an additional physical forcing affecting the displacement of the particles in the surface layer over time.

Specifically, daily fields of surface currents used for the simulation runs were from altimeter products (Absolute Geostrophic Velocities), as distributed by Copernicus marine environment monitoring service (CMEMS, http://marine.copernicus.eu/). Dates considered were 90-day long periods comprised between the 1^st^ of June and the 31^st^ of August for each year from 1997 to 2013, over the space domain 33–40°N and 8–20°E.

The influence of wind on surface circulation patterns was evaluated using a value-added 6-hourly global gridded (2.5° of latitude × 2.5°) analysis of ocean surface winds (NCEP Reanalysis data)^38^, available for download at http://www.esrl.noaa.gov/psd/. A detailed description on how the wind effect has been incorporated in the Lagrangian simulations is provided in the Supplementary Methods.

Daily surface current fields and wind data were included as external drivers for the whole duration of each simulation run. Finally, horizontal diffusion was also accounted for and incorporated in simulations as a random-walk process calculated from a uniform distribution^35,39^ using the GNOME default coefficient of 10^5^ cm^2^ s^−1^.

The simulation runs aimed at evaluating the distribution patterns of developing anchovy early stages (eggs + larvae) under the effect of hydrological and wind forcings during their planktonic phase^40^. The duration of the simulation runs was fixed at 28 days, the age at which anchovy larvae are considered able to swim fast enough to influence their horizontal motion within the current field^41,42^. Actually, during most of the larval life stages, the speed and the duration of swimming episodes are very limited^43^ and the energetic costs linked to the locomotory activity are very high due to the viscous environment in which frictional forces dominate^44,45^. So, despite fish larvae may have an active role on dispersal processes^46^, in general they can be considered with a good accuracy as passive particles drifted by the currents occurring in the upper layers^40^. Our assumption is also in agreement with findings by Faillettaz *et al*., who found a more efficient (coastward) larval behavior in species characterized by a relatively short pelagic larval duration (PLD = 13–18 days) compared with the longer ones (PLD = 28–38 days), such as the European anchovy^46^.

**Figure 2:**
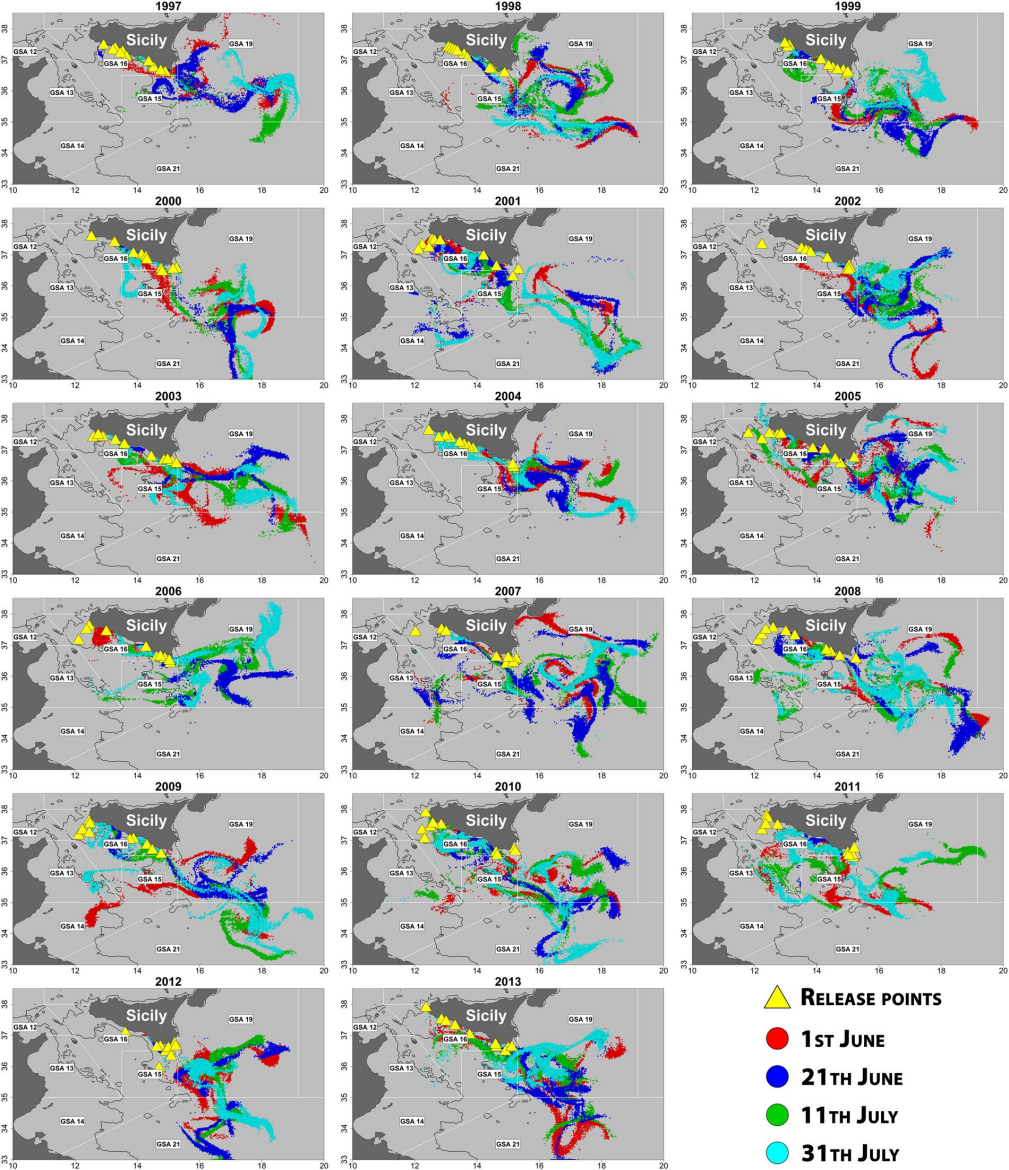
Final positions of particles released in each simulation run (duration: 28 days) for Scenario 1, by year. Colors of points corresponds to the selected 4 different releasing dates (June 1^st^, June 21^st^, July 11^th^, July 31^st^) within each year. Yellow triangles represent the positions (variable by year) of releasing starting points for particles (i.e., the stations scoring the 10 top highest anchovy eggs densities in #/m^3^ in each summer survey, from 1997 to 2013; see also Fig. 1).

Two simulation scenarios were adopted. In the first scenario (Scenario 1), for each one of the 17 summer surveys carried out over the period 1997–2013, the 10 most important stations in terms of anchovy egg concentrations were selected and considered as representative of the spatial distribution of the main yearly spawning grounds in the study area, so accounting for their interannual variability (Figs. 1 and 2). Their locations were used as releasing points for Lagrangian simulation runs (1,000 particles for each of the 10 stations).

**Figure 3:**
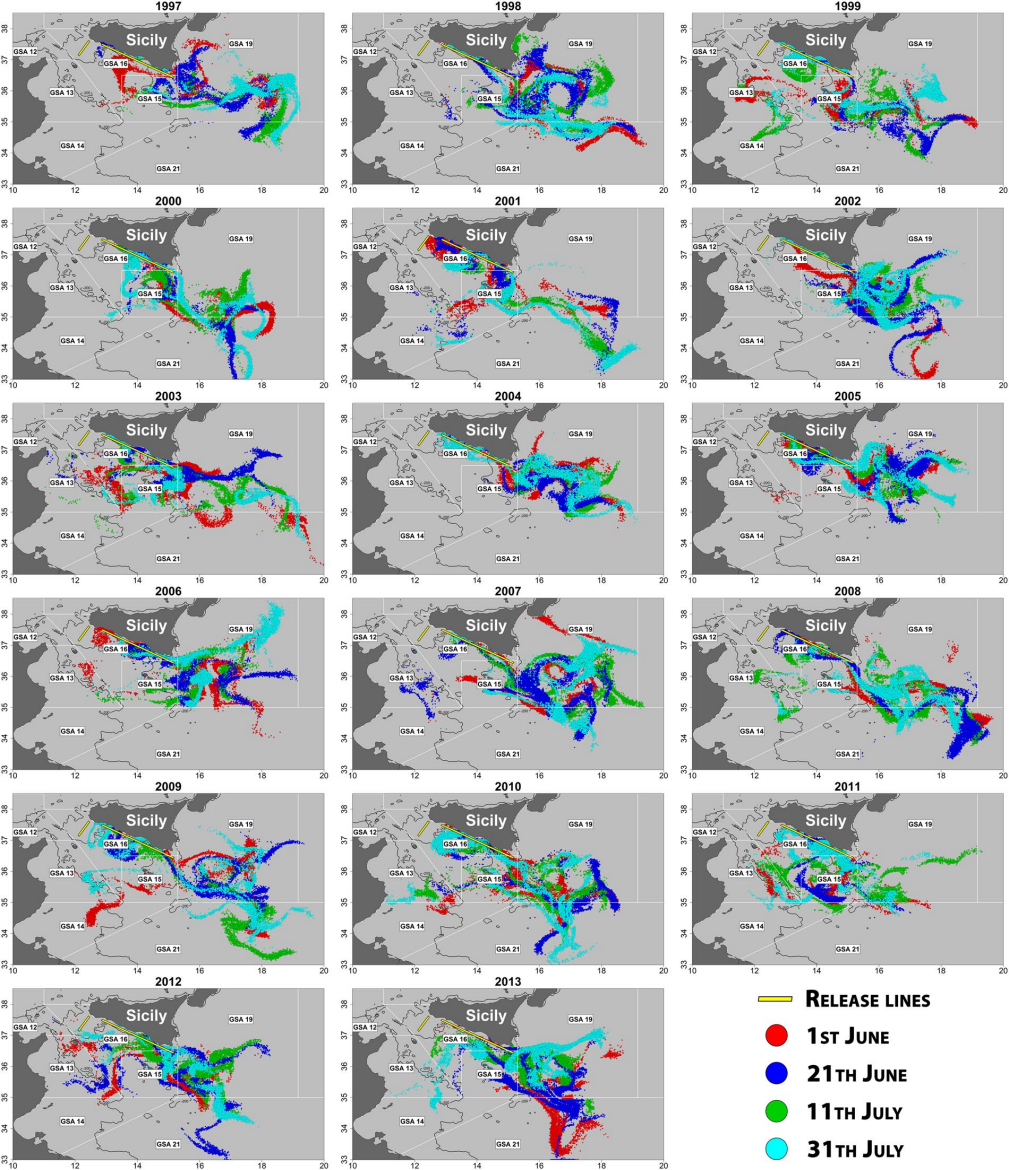
Final positions of particles released in each simulation run (duration: 28 days) for Scenario 2 by year. Colors of points corresponds to the selected 4 different releasing dates (June 1^st^, June 21^st^, July 11^th^, July 31^st^) within each year, whereas yellow lines represent the (fixed) positions of releasing starting points, placed on the main spawning grounds as detected by Basilone *et al*. (2013).

In the second scenario (Scenario 2), a uniform eggs distribution was applied, taking into account information about the anchovy spawning behavior in the study area^22^. Specifically, particles were released from two fixed transects positioned over the continental shelf, in numbers proportional to their lengths (2,700 points and 7,300 points, respectively; see Fig. 3).

In both simulation scenarios and for each year of time series, 4 different releasing dates were selected and used, at constant intervals 20 days apart (i.e.: June 1^st^, June 21^st^, July 11^th^ and July 31^st^). This choice was made in order to account for the expected effect of the intra-annual variability in the main physical factors (surface current and wind) driving the distribution of particles (representing anchovy pre-juvenile stages) in the study area over the considered period (June-July).

The geographical positions of released particles at the end of simulation runs were determined and classified in relation to the bottom depth (shelf/slope areas) and to the subdivision of Mediterranean waters into the Geographical Sub-Areas (GSAs) adopted by the General Fisheries Commission for the Mediterranean (GFCM). Information about local bathymetry, as extracted from ETOPO1 database, 1 Arc-Minute Global Relief Model, hosted on the NOAA website was used to this aim^47,48^.

In both the adopted simulation scenarios, the effect of wind on larval advection was considered incorporating information about the expected vertical distribution of anchovy larvae in the water column. More detailed information about the handling of wind data are given in Supplementary Material.

### Larval retention indices (LRIs)

The Larval retention indices (LRIs) adopted in this study are based on the total number of particles whose final positions at the end of the simulation runs occurred over the continental shelf (bottom depth < 200 m) area of GSA 16 (“South of Sicily”).

The rationale of this choice is that anchovy potential juvenile habitat is characterized by highly productive inshore waters^23,25^, where the environmental conditions are expected to be more favorable for the survival and development of early life stages. Different LRIs were constructed, integrating information from the four releasing dates available for each year of the time series (1997–2013) in both scenarios. Specifically, for each of the releasing dates and for each year included in the analysis, LRIs were evaluated singularly or averaged as groups of contiguous dates (Scenario 1: 3 groups of two dates, 2 groups of three dates, and finally 1 group with all dates, this last one covering the entire peak spawning period; Scenario 2: 1 group of two dates, 1 group covering the entire peak spawning period).

### The remote sensing dataset

Remote sensing data on sea surface temperature (SST) and chlorophyll-a concentration (CHL-a) collected during the spawning period and in the immediately following period (hereafter in the manuscript indicated as “post-spawning” period) have been used in this study. These two parameters, more relevant for growth and survival processes^21,49,50^, were considered as indicative of the environmental conditions experienced by the anchovy population in the study area during the planktonic and pre-recruitment stages. Monthly-mean SST (0.04° × 0.04° of spatial resolution) and CHL-a (0.01° × 0.01° of spatial resolution) data were firstly obtained from satellite products (L4, gap-free) made available by the Copernicus programme (http://marine.copernicus.eu/). Specifically, for each year over the period 1997–2013, average SST and CHL-a concentration values over the continental shelf of GSA 16 during the anchovy peak spawning (June-August, year *t*) and post-spawning (from September, year *t* to January, year *t* + 1) periods were calculated. These environmental factors were then used as independent variables in the modelling approaches aimed at predicting the biomass of the anchovy population at year (*t* + 1).

### The statistical models

The hypothesis of linear correlation between LRIs at year (*t)* and the recruitment success at year (*t* + 1) was firstly tested. Regressands were both the yearly anchovy biomass and the variable “biomass + catch” previously defined. The multiple hypothesis testing issue was also taken into account by using the highly conservative criterion based on Bonferroni correction^51^.

Further statistical modeling approaches included the Generalized Linear Models (GLMs) and the Generalized Additive Models (GAMs).

The distribution of response variables has been evaluated using Shapiro-Wilk normality test. Gaussian distribution and an “identity” link between the response variable and the systematic part of the model was considered in all models.

Concerning the GAMs, the potential non-linear relationships between covariates and the dependent variables were investigated by cubic regression splines. The possible issue of multicollinearity prior to model selection was also checked^52^. All LRIs were significantly correlated with the dependent variables, but only the one that allowed the estimation of the best model in terms of AIC was selected for the subsequent analysis.

Other statistical models were implemented for exploring the cumulative effect of the selected LRI and the environmental conditions occurring during the spawning (SSTspawn and CHLspawn) and the post-spawning period (SSTpost and CHLpost) on the two response variables considered.

Finally, all models were runs both including and excluding the first year of the time series (1997). The rationale was to verify its impact on the analysis of the egg concentration data obtained from the 1997 ichthyoplankton survey, which were considered potentially biased by the exploratory character of this first survey in the study area.

Akaike’s Information Criterion (AIC) was used for models’ comparison and selection^53^. Statistical analyses were implemented using *Statistica for Windows* software (v. 10) and the package “*mgcv*” of R software, version 3.3.2^54^.

## Results

### The effect of surface circulation (larval retention indices)

Figure 1 shows the density distributions of anchovy eggs (in #/m^3^) collected in the study area for each of the 17 summer surveys considered in the present study. The locations of the stations scoring the 10 top highest anchovy eggs densities over the continental shelf areas of GSA16 by year highlight the inter-annual spatial variability of main spawning grounds over time. Generally, the spawning grounds are displaced off the southern coast of Sicily, mainly at bottom depth in the range 50–100 m. However, in some years (e.g., in 2001 and 2010) spawning activity mainly occurred on the continental shelf area located in the NW side of the study area (Adventure Bank).

**Figure 4:**
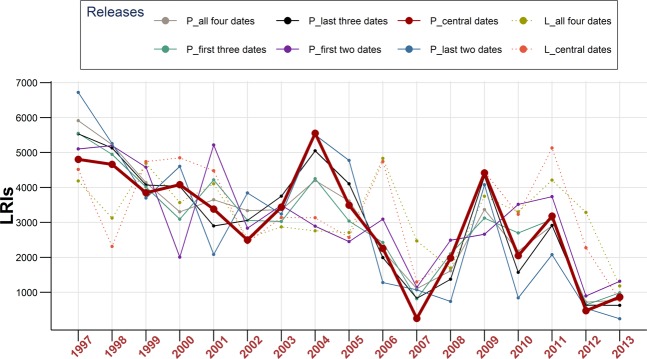
Interannual fluctuations of estimated larval retention indices (LRIs) for different groupings among the adopted releasing dates. For Scenario 1 (space dependent, based on the estimated main yearly spawning grounds areas; time series depicted with continuous lines), average indices for all the four dates and for all combinations with two or three dates grouped and averaged are shown (denoted with a starting “P” in the legend)). For Scenario 2 (fixed releasing locations for all years, uniformly distributed over the continental shelf of the study area; time series depicted with dashed lines), only average indices for all releasing dates grouped together and for “central dates” are shown. “Central dates” are 21^st^ June and 11^th^ July, and the corresponding LRI in Scenario 1 is evidenced with a thicker line. See text for further details.

The final particle distributions resulting from the two simulation scenarios described above are shown in Figs. 2 and 3. The yearly pattern distributions show a dominant Eastward advection in both scenarios, as expected due to the direction of main physical forcings (surface current and wind). However, inter-annual and intra-annual variations are quite evident. This variability affects the yearly values of the adopted larval retention (pre-recruitment) LRIs, which are somewhat correlated each other (Fig. 4), but with significant deviations from the general pattern depending on the releasing dates (e.g., in Scenario 1 see values for years 2000–2001, 2004–2005 and 2010–2011). However, it is worth noting that yearly deviations in Scenario 2 appear to have a quite different pattern compared to fluctuations observed in Scenario 1.

The difference between Scenario 1 and Scenario 2 are reflected in the relationships among yearly LRIs and biomass estimates (integrated or not by fish production) as reported in Table 1. Highly significant correlations were obtained in Scenario 1 using as independent variable the average LRI obtained assembling data from the two “central” releasing dates (June 21^st^ and July 11^th^; see Table 1). The spatial distribution of particles generating this LRI can be found in the Supplementary Figures S1 (Scenario 1) and S2 (Scenario 2).

**Table 1:** Linear correlation coefficients among LRIs and response variables “biomass” and “biomass + catch”, for Scenario 1 (LRI1) and Scenario 2 (LRI2), and for periods 1997–2013 and 1998–2013. LRI1_4 and LRI2_4: mean of all four releasing dates (June 1^st^, June 21^st^, July 11^th^, July 31^st^).

		Scenario 1						Scenario 2	
		LRI1_4	LRI1_3t	LRI1_3b	LRI1 _2c	LRI1_2b	LRI1_2t	LRI2_4	LRI2_c
1997–2013	Biomass	**0.521**	**0.649**	**0.515**	**0.723**	0.261	**0.580**	0.270	0.437
		p = 0.032	p = 0.005	p = 0.035	p = 0.001	p = 0.312	p = 0.015	p = 0.295	p = 0.080
	Biomass + catch	0.449	**0.591**	0.468	**0.704**	0.208	**0.512**	0.195	0.395
		p = 0.071	p = 0.013	p = 0.058	p = 0.002	p = 0.423	p = 0.036	p = 0.453	p = 0.117
1998–2013	Biomass	**0.701**	**0.780**	**0.677**	**0.815**	0.348	**0.752**	0.320	0.491
		p = 0.002	p = 0.000	p = 0.004	p = 0.000	p = 0.186	p = 0.001	p = 0.227	p = 0.054
	Biomass + catch	**0.675**	**0.762**	**0.678**	**0.833**	0.328	**0.729**	0.266	0.474
		p = 0.004	p = 0.001	p = 0.004	p = 0.000	p = 0.215	p = 0.001	p = 0.319	p = 0.064

LRI1_3t: last three dates (June 21^st^, July 11^th^, July 31^st^). LRI1_3b: first three dates (June 1^st^, June 21^st^, July 11^th^). LRI1_2c and LRI_2c: “central dates” (June 21^st^, July 11^th^). LRI1_2b: first two dates (June 1^st^, June 21^st^). LRI1_2t: last two dates (July 11^th^, July 31^st^). Significant results (p < 0.05) are in bold, in bold and underlined the most significant results satisfying the Bonferroni’s correction (see text).

**Figure 5:**
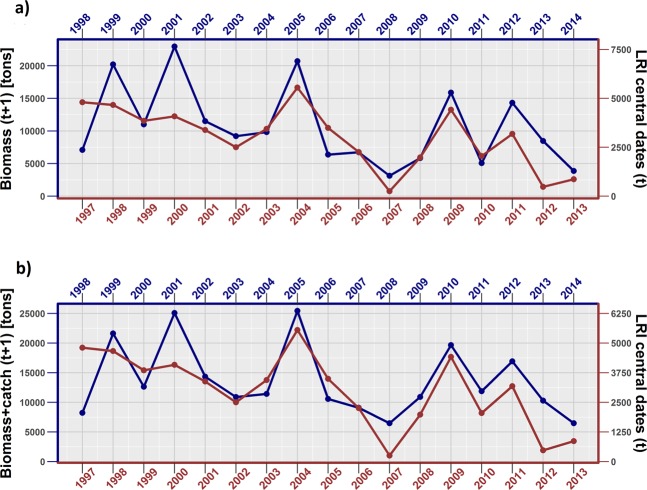
Trends in larval retention index (LRI) on Scenario 1 calculated on “central” dates (June 21^st^–July 11^th^) for the period 1997–2013, compared to (**a**) anchovy acoustic biomass estimates one year ahead (from 1998 to 2014) and (**b**) anchovy acoustic biomass estimates one year ahead (from 1998 to 2014) integrated with landings (see text for details).

Quite similar results were obtained switching the regressand “biomass” with the variable “biomass + catch”. Figure 5a,b shows the trends in the yearly LRI (from 1997 to 2013) calculated for central dates under Scenario 1, compared to the anchovy biomass and to the biomass + catch variable one year ahead (from 1998 to 2014).

However, it is worth noting that, for both the dependent variables used in the analysis, even higher correlations values were obtained removing the first year (1997) from the time series (Table 1).

Results were still significant even taking into account the multiple hypothesis testing issue (Miller, 1981), at least when adopting as regressor the LRI calculated over the central dates. Conversely, none of the estimated correlations were significant for Scenario 2 (p > 0.05 in all cases), even though higher values were always obtained using the central dates (Table 1).

When regressing time series, a possible problem that needs to be controlled is the non-stationarity of the variables included in the analysis. Actually, it is well known that spurious correlations between variables may arise in presence of common trends. In our case, all the independent variables (LRIs) used in the simple linear regression analysis have a significant decreasing trend (a decreasing trend is also present in the dependent variables, though it is not significant). So, in order to verify the effect of these trends on the detected linear relationships, additional regression models were estimated considering (or not) the year 1997 and removing the linear trends from both independent (LRI according to Scenario 1 and considering “central dates”) and depended variables (biomass or biomass + catch) before the analysis. The best linear correlation coefficients were estimated by models that exclude 1997 from the time series (Biomass ~ LRI: r = 0.7242, p-value = 0.001; Biomass + catch ~ LRI: r = 0.7831, p-value < 0.001). However, significant correlation terms emerged also from models that include this year and consider biomass (r = 0.6655, p-value = 0.004) or biomass + catch (r = 0.7145, p-value = 0.002) as dependent variables.

In summary, results show that the percentage of variance in yearly anchovy biomass explained by surface circulation in the study area is in the range 44–51% when considering the entire time series and up to 61–70% when excluding data of first year from the time series.

GAMs confirmed the linear relationship among variables and highlighted the same patterns evidenced by linear regression models.

### The cumulative effect of LRI and other environmental factors

**Figure 6:**
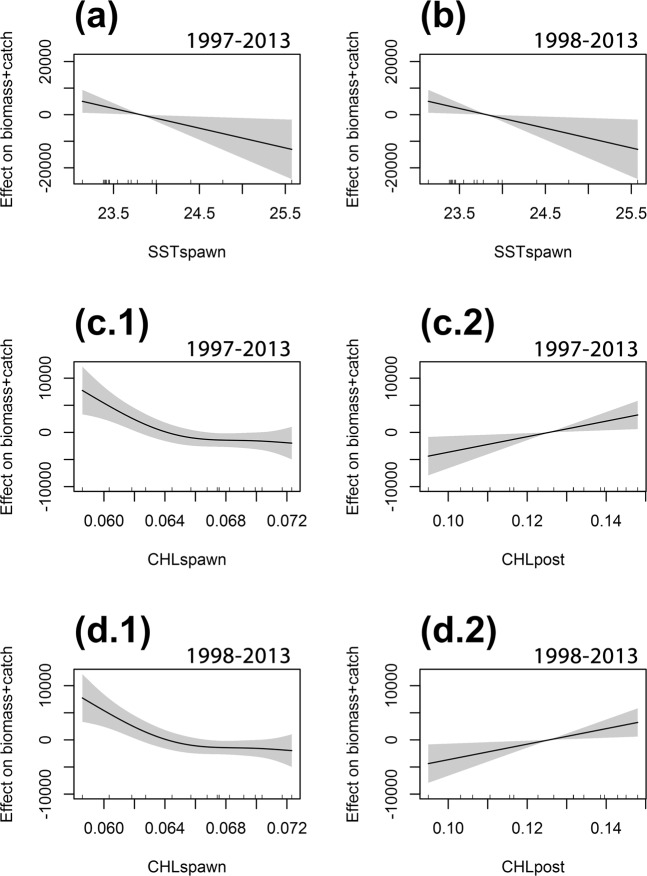
Trend of the significant cubic regression splines implemented in the GAMs described in Table 2. Only significant environmental factors are considered. Upper panels refer to models not including the LRI index among the factors, with (**a**) using the entire available time series and (**b**) excluding the first year (1997 for the environmental factors and 1998 for the anchovy acoustic biomass estimate. Panels c1,2) and d1,2) refer to models that include the LRI index, again with (**c**) panels using the entire available time series and (**d**) panels excluding the first year. See text for further details.

GLMs and GAMs were initially used in order to investigate the relationship between the anchovy biomass (or biomass + catch) and some selected environmental factors characterizing the potential anchovy habitat during the spawning period (June–August, parameters SSTspawn and CHLspawn) and during the juvenile growth (September-January, parameters SSTpost and CHLpost)^49^. Moreover, the same statistical methods were applied including the LRI based on the central dates under Scenario 1 among the regressors. All models were run with or without the inclusion of the first year of the time series (1997). Results are summarized in Table 2 and in Fig. 6.

**Table 2:** Outcomes of Generalized Linear Models (GLMs) and Generalized Additive Models (GAMs).

Model	Dataset	Dependent variable	Independent variables	sign terms (estimate)	p-value	Dev. Expl.	AIC
GLM	1997–2013	biomass	SSTspawn + CHLspawn + SSTpost + CHLpost	non significant terms			
GLM	1997–2013	biomass + catch	SSTspawn + CHLspawn + SSTpost + CHLpost	non significant terms			
GLM	1998–2013	biomass	SSTspawn + CHLspawn + SSTpost + CHLpost	non significant terms			
GLM	1998–2013	biomass + catch	SSTspawn + CHLspawn + SSTpost + CHLpost	non significant terms			
GAM	1997–2013	biomass	s(SSTspawn) + s(CHLspawn) + s(SSTpost) + s(CHLpost)	non significant terms			
GAM	1997–2013	biomass + catch	s(SSTspawn) + s(CHLspawn) + s(SSTpost) + s(CHLpost)	SSTspawn (Fig. 6.a)	0.043	63.62%	321.84
GAM	1998–2013	biomass	s(SSTspawn) + s(CHLspawn) + s(SSTpost) + s(CHLpost)	non significant terms			
GAM	1998–2013	biomass + catch	s(SSTspawn) + s(CHLspawn) + s(SSTpost) + s(CHLpost)	SSTspawn (Fig. 6.b)	0.043	63.62%	321.84
GLM	1997–2013	biomass	LRI + SSTspawn + CHLspawn + SSTpost + CHLpost	LRI (2.871)	0.001	80.33%	311.94
				CHLspawn (−6.512e + 05)	0.038		
GLM	1997–2013	biomass + catch	LRI + SSTspawn + CHLspawn + SSTpost + CHLpost	LRI (2.867)	0.001	82.05%	309.51
				CHLspawn (−6.078e + 05)	0.037		
GLM	1998–2013	biomass	LRI + SSTspawn + CHLspawn + SSTpost + CHLpost	LRI (2.871)	0.001	80.33%	311.94
				CHLspawn (−6.512e + 05)	0.038		
GLM	1998–2013	biomass + catch	LRI + SSTspawn + CHLspawn + SSTpost + CHLpost	LRI (2.867)	0.001	82.05%	309.51
				CHLspawn (−6.078e + 05)	0.037		
GAM	1997–2013	biomass	LRI + s(SSTspawn) + s(CHLspawn) + s(SSTpost) + s(CHLpost)	LRI (2.8663)	0.001	85.70%	308.91
GAM	1997–2013	biomass + catch	LRI + s(SSTspawn) + s(CHLspawn) + s(SSTpost) + s(CHLpost)	LRI (2.9253)	0.005	91.90%	300.66
				CHLspawn (Fig. 6.c1)	0.035		
				CHLpost (Fig. 6.c2)	0.038		
GAM	1998–2013	biomass	LRI + s(SSTspawn) + s(CHLspawn) + s(SSTpost) + s(CHLpost)	LRI (2.8663)	0.001	85.70%	308.91
GAM	1998–2013	biomass + catch	LRI + s(SSTspawn) + s(CHLspawn) + s(SSTpost) + s(CHLpost)	LRI (2.9253)	<0.001	91.90%	300.66
				CHLspawn (Fig. 6.d1)	0.035		
				CHLpost (Fig. 6.d2)	0.038		

In GAM, the use of s() stands for the application of the cubic regression spline as a smooth term. When significant terms (sign. terms) were detected, the model regression coefficients (estimate) and the associated p-value are shown, as well as the deviance explained and the AIC index, as an indicator of the model fitting performance.

The selected environmental factors (SSTspawn, CHLspawn, SSTpost and CHLpost) alone did not produce significant effects in the multiple linear regression approach. On the other hand, GLMs including the selected LRI among considered factors highlighted both the significant positive effect of this index and the negative contribution of the CHL-a concentrations. The fitting performance in terms of deviance explained and AIC improved when the variable “biomass + catch” was used as dependent variable. Conversely, no improvements in the fits were detected when excluding the first year of the time series from the analysis.

GAMs confirmed these findings and allowed to achieve the best fitting results. The application of the cubic regression splines on the selected environmental factors alone did not show significant relationships with the regressand “biomass”. However, SST during the spawning period (SSTspawn) was found negatively related to the regressand “biomass + catch” (Table 2 and Fig. 6a,b), even though the test statistics was barely significant in this case (p = 0.043).

When the LRI factor was included in the GAMs, the fitting performance dramatically improved (with deviance explained ranging from 85.7% to 91.9%). LRI was the most significant term in all models, while the CHL-a concentration term resulted significant only when “biomass + catch” was used as dependent variable. In particular, a positive linear relationship emerged with the CHL-a concentration during the post-spawning period (CHLpost), while the CHL-a concentration occurring during the spawning period (CHLspawn) showed a non-linear trend, with a negative relationship only at lower values (CHL-a < 0.065 µg/l) (Fig. 6c,d). Similarly to what observed using the linear (GLM) approach, no differences emerged when excluding from the analysis the first year of the time series.

## Discussion and Conclusions

The obtained results are in accordance with previous studies showing the importance of factors other than fishing pressure, namely bottom-up processes induced by environmental variability, in affecting the interannual fluctuations on the standing stock biomass of small pelagic species^3,6,55^. Indirectly, in our study this feature appears to be confirmed by the similar model outputs obtained with the two adopted dependent variables (“biomass” and “biomass + catch”). In addition, the available time series for anchovy landings is not correlated with anchovy biomass.

Most of the observed variance was explained by the adopted LRI. The comparison of the two simulation scenarios suggests the importance of the yearly location of the main spawning grounds, variable across years, in modulating the recruitment strength and, definitely, the fish standing stock biomass available for the exploitation of local fisheries. In fact, significant correlations were only observed when using the LRIs of Scenario 1, which implies the incorporation of prior knowledge on the spawning grounds.

In general, our results support larval retention hypothesis in evidencing the importance for larval production of being retained in natal coastal spawning areas in order to favor the recruitment success^56–58^.

In addition, our findings confirm the importance of spatially-based approaches aiming at reproducing the dynamics of the recruitment of small pelagic fish.

Our results are in line with the findings of previous studies that indicated the central role of ocean circulation in driving recruitment strength and, definitely, resource abundance^59,60^. These last studies, focused on the recruitment of the European anchovy in the NW Mediterranean Sea, used Spatially-explicit individual-based Models (SEIMBs) to provide operational information that can be used to deliver early predictions of fish abundance indices and improved spatio-temporal management strategies. Actually, the impact of unfavorable transport to offshore waters is very similar to the pattern evidenced by our simulation approach in the Northern SoS. In particular, in Scenario 1 on average just about 30% of larvae (particles) were transported to coastal areas, with a quite high interannual variability (CV = 52%).

Incidentally, results for Scenario 2 show a higher percentage of larval retention in shelf areas (32%), but a much lower variability (CV = 36%), stressing the importance of interannual variability in favoring the success of recruitment processes. Therefore, as well as already shown for the anchovy population in the NW Mediterranean^59^, prior knowledge of the initial spawning grounds may strongly affect recruitment estimates. In addition, in both areas the inter-annual fluctuations in anchovy recruitment (which in the study area is also a proxy for total biomass) are correlated with the success of larval retention processes, despite the quite low observed retention rate^60^. However, though SEIBMs are very powerful tools in predicting spatio-temporal patterns for recruitment, they are much more data (and analysis) demanding compared to the simpler approach adopted in this study. In our case, data needs for getting predictions on recruitment strength (and, for anchovy in Northern SoS, also on the biomass of the entire fish population) mainly concern the basic information to build the LRIs, i.e. the anchovy eggs distribution and concentrations (obtained from surveys) and the satellite-based daily surface currents during the anchovy peak spawning period in the study area. It is worth noting that our results would allow for an early estimation anchovy biomass one year in advance compared to the availability of hydroacoustic-based estimates, which are typically delivered after several months following the survey at sea. This represents a very important and relevant product in support to fisheries management.

Another important remark is that the observed relationships should be considered at least as partially independent on the measured average intensity of yearly spawning activity, as inferred by the absolute fish egg densities estimated through the yearly summer surveys. Actually, this last information was used in the approach herein adopted just for determining the locations of releasing starting points for particles of Scenario 1, variable across years. In general, the importance of advection of anchovy fish larvae by the surface currents is evidenced in both scenarios. However, the better results obtained within Scenario 1 compared to Scenario 2, which did not produce significant relationships, underline the role that regular plankton sampling surveys may have in delivering early predictions of the anchovy standing stock biomass. In fact, scientific surveys represent the only way to get information on the interannual variability in the distribution of the main spawning grounds within the study area, and this knowledge is essential for the construction of the LRIs used in this study.

Another indirect result of the observed correlation patterns between LRIs and anchovy biomass is about the timing for anchovy spawning that can be generally considered more favorable for the success of recruitment processes in the Northern SoS. In fact, the adopted time span (June-July) for particles releasing in the Lagrangian simulation runs was wide enough to catch a subset of dates (in this study, the “central” ones) that are the best ones in terms of probability of getting more accurate predictions of recruitment (and population biomass) strength. In addition, the consistent correlation results obtained using the different LRIs time series also suggest that intra-annual movements of the spawning stock within the study area are most probably quite limited. Namely, an important assumption for the validity of the adopted approach is that the main spawning grounds are relatively stable within the spawning season, even though only the carrying out of multiple ichthyoplankton surveys during the reproductive period would allow for verifying this hypothesis.

GLM and GAM modeling approaches used in this study were also informative on the limited contribution that other environmental factors such as SST and CHL-a concentration may have in improving the accuracy of anchovy biomass predictions. Actually, even though their contribution in the increasing the explained deviance was low, the detected significant patterns are consistent with the present knowledge on the ecology of the anchovy population in the Northern side of the SoS. In particular, the detected significant negative impact of SST during the spawning period on anchovy biomass is consistent with what is known about the negative effect that high temperature regimes may have on eggs production^22^. In addition, the positive relation between CHL-a concentration during the post-spawning (or pre-recruitment) period and anchovy biomass one year ahead is consistent with the expected higher survival rates for juvenile stages during the pre-recruitment phase, when the more favorable feeding conditions are able to support faster growth rates^24,28,49^.

However, further research efforts are needed to investigate the role of other physical forcings, such as temperature and/or chlorophyll concentration, in affecting the mortality rates of advected anchovy larvae (which would eventually influence the magnitude of larval retention indices as calculated in the present paper) and even the survival of juvenile stages. Moreover, the influence of the larval swimming ability on the dispersion of the early life stages represents an interesting research question that could be further investigated. Although this study showed that the passive larval dispersion in the first 28 days during the spawning season in one year represents the main factor affecting the anchovy stock biomass of the following year, we cannot exclude that a potential effect of the active swimming behavior in particular by late larval stages could improve larval retention (and recruitment success), as showed for other demersal species^46^.

Other relevant fields of investigation are about the factors affecting the general surface circulation and the patterns that are able to favor larval retention processes in Northern SoS. This topic, which is outside the scope of the present study, is worth of further research effort because it would permit to speculate on possible future scenarios for this fish population in relation to other physical forcings, including the increasing trend in the temperature regime of Mediterranean waters.

Finally, the same approach used in this paper for anchovy in Northern SoS could be applied to other small pelagic fish species such as European sardine (*Sardina pilchardus*) and round sardinella (*Sardinella aurita*) in the same study area or in other ecosystems where the variability of the environmental conditions may strongly impact on the fate of reproduction offspring.

## Supplementary information

SUPPLEMENTARY INFO.

## Data Availability

The datasets generated during and/or analyzed during the current study are available from the corresponding author on reasonable request.
