# Supplementary material for: General surface circulation controls the interannual fluctuations of anchovy stock biomass in the Central Mediterranean Sea
Source: Sci Rep. 2020 Jan 31;10:1554. doi: 10.1038/s41598-020-58028-0 (PMC6994607; doi:10.1038/s41598-020-58028-0)

Supplementary material to

"General surface circulation controls the interannual fluctuations of anchovy stock biomass in the Central Mediterranean Sea"

Bernardo Patti, Marco Torri* and Angela Cuttitta

Institute of Anthropic Impacts and Sustainability in marine environment, National Research Council of Italy, 91021 Campobello di Mazara, Italy

*Corresponding author. Email address: marco.torri@ias.cnr.it

Supplementary Methods. Handling of wind data in the Lagrangian simulations.

Within the Lagragian simulation runs, the effect of wind on larval advection incorporated information about the expected vertical distribution of anchovy larvae in the water column.

Wind is typically included in particle-tracking models assuming that the surface wind-induced current (windage effect) is about 3% of the wind speed (Pugh, 1987; Stolzenbach et al., 1977), declining logarithmically to zero at approximately a depth generally assumed to be 20 m (Elliott, 1986). Previous studied showed that the bulk of the anchovy larval stages is concentrated in the surface layers of the water column, above the thermocline (Palomera, 1991; Coombs et al., 2003; Ospina-Álvarez et al., 2012b; Olivar et al., 2001, 2014). Therefore, a reference depth range of 1-10 m was adopted for the following calculation of the effect of wind forcing, as an additional factor contributing to larval advection simulated in the GNOME environment.

The wind-induced current at depth *x* (in meters) can be estimated using the following equation (Pugh, 1987):

$$u_x = u_0 - \frac{u^*}{k} \ln\left(\frac{x}{z_0}\right)$$

where u_0 is the surface wind-induced current, k = 0.4 is the von Karman constant, u^* is the friction velocity that can be estimated as 0.0012*W, with W being the wind speed 10 m above the sea surface, and finally z_0 is the sea surface roughness length, fixed at 0.001 m. Taking into account the above formulation, the windage effect (i.e., the parameter used within GNOME accounting for the movement of particles induced by the wind) was set in the range 0.93-0.23%. These values represent the wind-induced current at the depths of 1 m and 10 m in terms of fractions of wind speed over the sea surface.

References

1. Coombs, S. H., Giovanardi, O., Halliday, N. C., Franceschini, G., Conway, D. V. P., Manzueto, L., Barrett, C. D. & McFadzen, I. R. B. (2003). Wind mixing, food availability and mortality of anchovy larvae *Engraulis encrasicolus* in the Northern Adriatic Sea. *Marine Ecology Progress Series*, 248, 221–235.
2. Elliott, A. J. (1986). Shear diffusion and the spread of oil in the surface layers of the North Seas. *German Journal of Hydrography*, 39, 113-137.

3. Olivar, M.P., Salat, J., & Palomera, I. (2001). Comparative study of spatial distribution patterns of the early stages of anchovy and pilchard in the NW Mediterranean Sea. *Marine Ecology Progress Series*, 217, 111–120.

4. Olivar, M. P., Sabatés, A., Alemany, F., Balbín, R., de Puelles, M. L. F., & Torres, A. P. (2014). Diel-depth distributions of fish larvae off the Balearic Islands (western Mediterranean) under two environmental scenarios. *Journal of Marine Systems*, 138, 127–138.

5. Ospina-Álvarez, A., Parada, C., & Palomera, I. (2012). Vertical migration effects on the dispersion and recruitment of European anchovy larvae: From spawning to nursery areas. *Ecological Modelling*, 231, 65-79.

6. Palomera, I. (1991). Vertical distribution of eggs and larvae of *Engraulis encrasicolus* in stratified waters of the western Mediterranean. *Marine Biology*, 111, 37–44.

7. Pugh, D. T. (1987). Tides, Surges and Mean Sea Level. Chichester: Wiley.

8. Stolzenbach, K. D., Madsen, O. S., Adams, E. E, Pollack, A. M., & Cooper, C. K. (1977). A Review and Evaluation of Basic Techniques for Predicting the Behavior of Surface Oil Slicks. Cambridge: Rep. 22, Dep. of Civ. Eng., Mass. Inst. Of Technol.

Supplementary Figure S1. Final positions of particles released in the dates June 21st and July 11th of the years between 1997 and 2013 for Scenario 1. Yellow triangles represent the positions (variable by year) of releasing starting points for particles (i.e., the stations scoring the 10 top highest anchovy eggs densities in #/m3 in each summer survey, from 1997 to 2013).

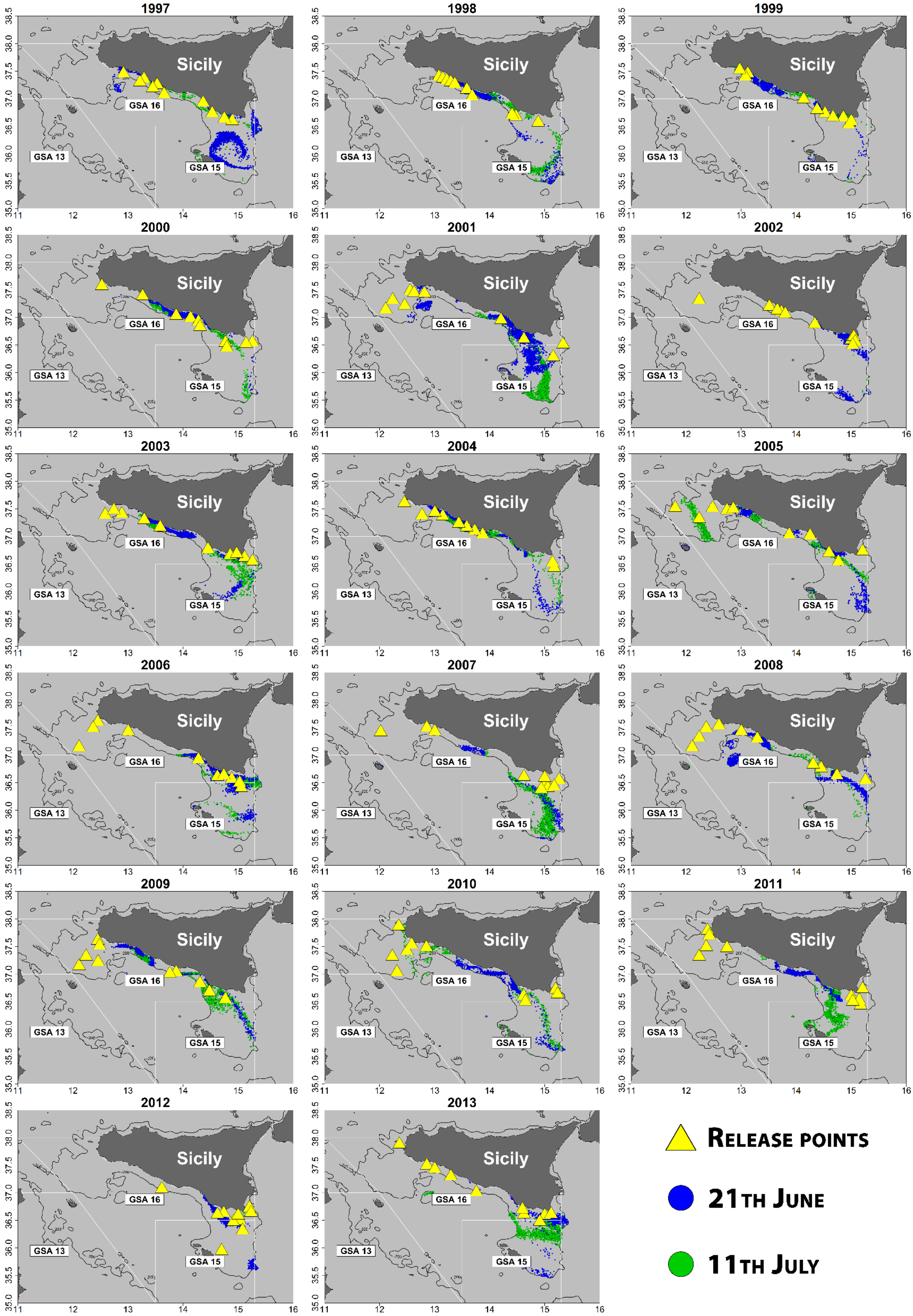

Supplementary Figure S2. Final positions of particles released in the dates June 21st and July 11th of the years between 1997 and 2013 for Scenario 2. Yellow lines represent the (fixed) positions of releasing starting points, placed on the main spawning grounds.

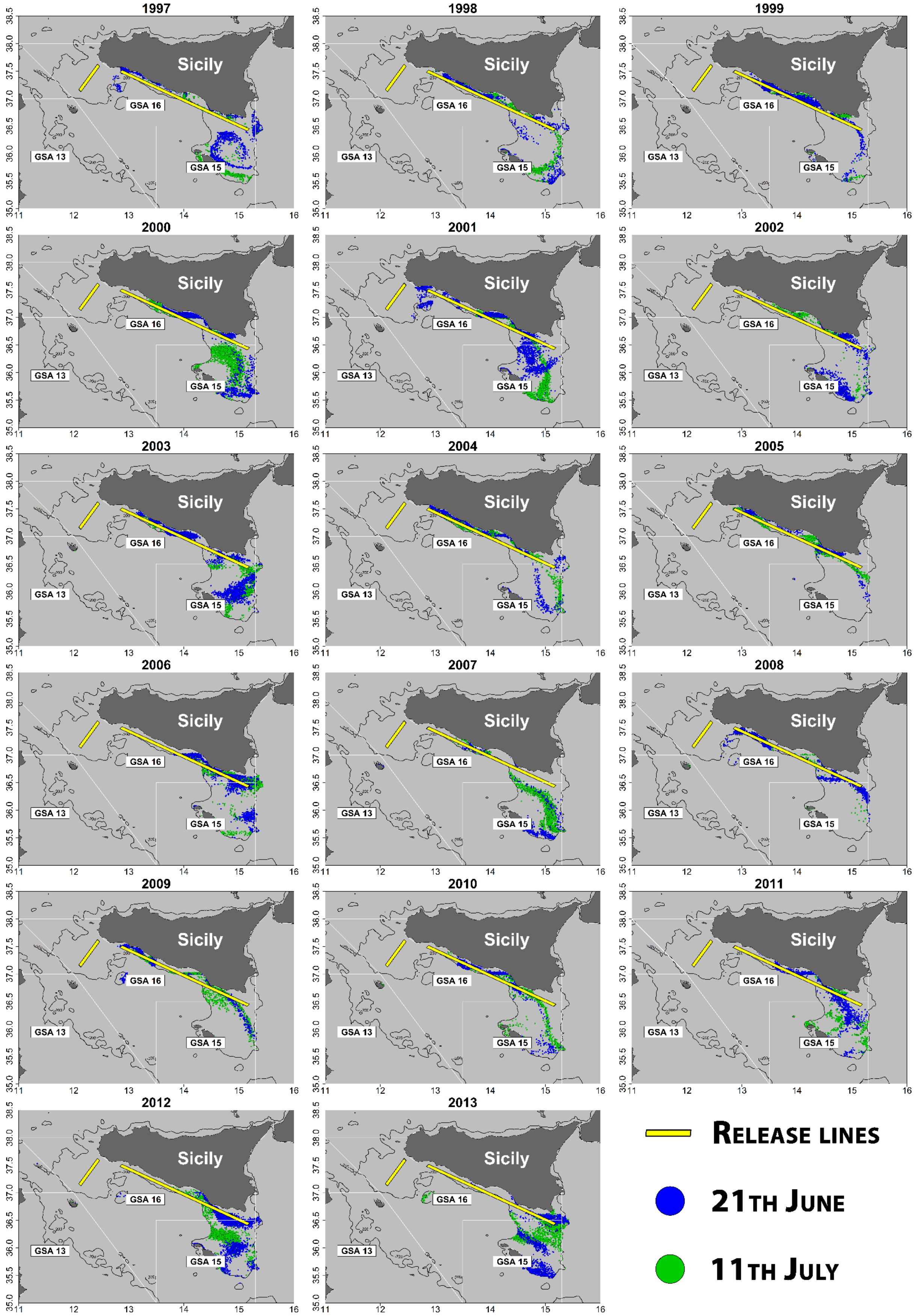

Supplement: Supplementary file 1 — SUPPLEMENTARY INFO. [file 41598_2020_58028_MOESM1_ESM.pdf]
